# *Allium ampeloprasum* var. Porrum (Alliaceae) Improves Metabolic and Reproductive Disorders Associated with Polycystic Ovary Syndrome in *Wistar* Rats

**DOI:** 10.1155/2024/8364343

**Published:** 2024-01-19

**Authors:** Alison Degrace Fofie Tedongmo, Marie Alfrede Mvondo

**Affiliations:** Research Unit of Animal Physiology and Phytopharmacology, University of Dschang, P.O. Box 67, Dschang, Cameroon

## Abstract

To provide scientific evidence of the efficacy of *Allium ampeloprasum* against female infertility, the effects of the aqueous extract of the said plant (AE) were evaluated in rats with letrozole-induced polycystic ovary syndrome (PCOS). AE was administered orally to PCOS rats at doses of 192, 384, and 768 mg/kg. The positive control was co-treated with clomiphene citrate (1 mg/kg) and metformin (200 mg/kg). Normal and negative controls received distilled water. The vaginal contents of rats were examined daily under a microscope before (7 days) and during treatment. Treatments were administered orally for 15 days, and then, 6 rats from each group were sacrificed for biochemical and histological analyses. The remaining rats were mated with males of proven fertility for 5 days. The daily examination of vaginal smears allowed the evaluation of fertility index. After parturition, additional fertility parameters were determined. Results showed that in PCOS rats, AE decreased body weight (*p* < 0.001), abdominal fat weight (*p* < 0.001), serum levels of LH (*p* < 0.001), testosterone (*p* < 0.001), total cholesterol (*p* < 0.05), and LDL cholesterol (*p* < 0.01). HDL cholesterol increased and atherogenic indices decreased (*p* < 0.001). The number of Graafian follicles and *corpora lutea* increased, while cystic (*p* < 0.001) and atretic (*p* < 0.05) follicles decreased. AE also decreased oxidative stress in the ovaries, restored the estrous cycle, induced uterine epithelial cell hypertrophy, and improved fertility. These effects were attributed to phenols, flavonoids, terpenoids, and anthocyanins present in AE. The overall results justify the traditional use of *A. ampeloprasum* against female infertility and suggest its potential use as a dietary supplement for PCOS patients.

## 1. Introduction

Infertility is considered a disease of the reproductive system characterized by the inability to achieve a clinical pregnancy after 12 months or more of regular unprotected sexual intercourse [1, 2]. 10–15% of reproductive-aged couples are affected worldwide [3]. This reproductive disease affects different areas of a couple's life as the ability to reproduce is closely linked to self-image, self-respect, and sexuality [4, 5]. Although male infertility contributes to about 50% of cases of global childlessness, infertility remains a woman's social burden [6]. The female reproductive function can be impaired by innate or acquired circumstances that affect the normal function of reproductive organs, illnesses, or psychological factors [4]. One of the most common diseases affecting the function of female reproductive organs is polycystic ovary syndrome (PCOS).

PCOS is an endocrine and metabolic disorder affecting 5–20% of women of childbearing age [7]. It is thought to be the most common cause of chronic hyperandrogenic anovulation and female infertility [8]. Metabolic disorders associated with PCOS include obesity, insulin resistance, dyslipidemia, and type 2 diabetes mellitus [7, 9]. Hormonal changes occurring in PCOS women include hyperandrogenism, high levels of luteinizing hormone (LH), and hyperinsulinemia [9–11]. Hyperandrogenism is an important criterion for the diagnosis of PCOS and plays a crucial role in the development and progression of PCOS [7]. Chronic elevated levels of androgens induce the overproduction of gonadotropin-releasing hormone (GnRH) and LH by the hypothalamus and the anterior pituitary, respectively [12]. In addition, hyperandrogenism stimulates pancreatic *β*-cells to release insulin [11]. The resulting hyperinsulinemia increases the bioavailability of androgens and insulin-like growth factor (IGF)-1 [13, 14]. IGF-1 and insulin potentiate the effect of LH on theca cells of the ovarian follicles to promote the production of androgens [15, 16]. High levels of insulin and IGF-1 also amplify the effect of LH on granulosa cells, causing premature differentiation, follicle growth arrest, anovulation, and cyst formation [17, 18].

Treatment for PCOS aims to relieve the clinical manifestations of PCOS and include antiandrogens, insulin sensitizers (e.g., metformin), or ovulation induction therapies (e.g., clomiphene citrate, gonadotropins, and laparoscopic ovarian drilling (LOD)) [19, 20]. Metformin and clomiphene citrate are often combined to increase ovulation and pregnancy rates [19, 21]. Gonadotropins are used when oral ovulation induction drugs fail to induce ovulation in patients with PCOS [19]. LOD and medically assisted procreation (MAP) are generally performed in infertile PCOS patients who have not had a favorable outcome with drug treatments [19, 22]. However, current treatment plans still have many shortcomings [19, 23] and some of them (gonadotropins and surgical interventions) are costly and time-consuming and their use requires intensive monitoring [19]. It is therefore necessary to continue to develop more effective and accessible alternatives for better management of PCOS and its complications.

Previous studies suggest that medicinal plants could be potential alternatives in the management of PCOS [24, 25]. Several Cameroonian herbal medicines were found to correct PCOS-related symptoms in rats [26–28]. These include *Phyllanthus muellerianus* [26], *Myrianthus arboreus* [27], and *Milicia excelsa* [28]. These herbal essences have been shown to decrease serum testosterone and LH levels and to restore ovarian function and fertility in PCOS rats [26–28]. To contribute to the popularization of the Cameroon pharmacopeia potentially effective against PCOS and associated infertility, we undertook to evaluate the effects of the aqueous extract of *Allium ampeloprasum* var. porrum (Alliaceae) in animals with PCOS. This plant, also called leek, is consumed in Cameroon as a vegetable or spice. It also has a medicinal virtue as it is traditionally recommended to cure female infertility [29]. Previous work has reported the hypoglycemic, lipid-lowering, and antioxidant effects of this plant in diabetic rats [30], suggesting its ability to improve physiological disorders associated with PCOS.

In the present study, the aqueous extract of *A. ampeloprasum* was chemically screened for the detection of the classes of compounds that are present in it, in particular those endowed with antioxidant, estrogenic, hypoandrogenic, and hypolipidemic properties. These include phenols, flavonoids, terpenoids, and anthocyanins. Indeed, the literature reports that phenolic compounds and terpenoids are endowed with anti-inflammatory properties [31]. Flavonoids are known to be estrogen-mimicking compounds [32, 33]. Polyphenols, such as flavonoids and tannins, were found to have an antihyperglycemic effect [34]. Soy isoflavones were also found to improve endocrine (decrease in serum testosterone levels) and metabolic (decrease in serum lipid levels) status in women with PCOS [35, 36]. Finally, anthocyanins were found to have antioxidant activities [37].

## 2. Materials and Methods

### 2.1. Plant Collection and Authentication

The fresh samples of *Allium ampeloprasum* were purchased from the Dschang food market (West Region, Cameroon) in December 2020 and the plant was authenticated at the Cameroon National Herbarium under the number 67469/HNC.

The aqueous extract of *A. ampeloprasum* was prepared following the method described by Edouard et al. [29]. Briefly, 20 large fresh leeks (3 kg) were boiled in 5 L of distilled water for 20 minutes. After sieving and filtration on Whatman No. 4 filter paper, the filtrate obtained was freeze-dried (−45°C) at the Institute for Medical Research and the Study of Medicinal Plant (Yaoundé, Cameroon), using a freeze dryer Christ Beta 1–8 LSCbasic (Osterode am Harz, Germany). Following this process, a total dry mass of 69.613 g of the aqueous extract of *A. ampeloprasum* was obtained and kept at 4°C in an airtight container until use.

### 2.2. Chemical Screening of the Aqueous Extract of *A. ampeloprasum*

To highlight the major classes of secondary metabolites of the aqueous extract of *A. ampeloprasum*, the analytical methods described by Harbone [38] were carried out.

The presence of phenolic compounds in the aqueous extract of *A. ampeloprasum* was detected by suspending 0.01 g of extract in 3 ml of ethanol. The mixture then received 3 drops of iron III chloride at 10% (V/V). At the end of this methodology, the appearance of a blue-violet or greenish color indicates the presence of phenols.

The detection of flavonoids was carried out using the Shinoda test. Briefly, 0.01 g of the extract was dissolved in 3 ml of methanol. The mixture was treated with 0.05 g of magnesium chips and 3 drops of concentrated HCl. The appearance of orange (flavones), red (xanthones), and pink (flavonols) colors indicate the presence of flavonoids. For the detection of chalcones and aurones, 0.01 g of extract was mixed with 3 ml of concentrated sulfuric acid and then stirred for 5 minutes. The presence of chalcones was characterized by the appearance of a red color, while aurones were marked by the appearance of a blue color.

Meyer test was used for the detection of alkaloids. Thus, 0.01 g of the extract was placed in a test tube in the presence of 3 ml of an aqueous solution of hydrochloric acid (50% V/V). The mixture was treated with 3 drops of “Meyer's reagent.” The formation of a white or yellowish color indicates the presence of alkaloids.

For the detection of saponins, 0.01 g of the extract was dissolved in 5 ml of distilled water and then boiled for 5 minutes. After cooling, each tube containing the dissolved extract was shaken vigorously for 30 seconds and then allowed to stand. The appearance of persistent foam with a height of more than 1 cm characterizes the presence of saponins.

The Liebermann–Burchard test was performed to detect the presence of triterpenoids and steroids. Thus, 0.01 g of the extract was dissolved in 3 ml of chloroform, and then, 3 ml of acetic anhydride acid was added and the mixture was cooled on ice for 3 minutes. Finally, a drop of concentrated sulfuric acid was added. The presence of triterpenes was confirmed by the appearance of a purplish red color and that of steroids was confirmed by the appearance of a blue or green color.

The presence of quinones was analyzed by dissolving 0.01 g of the extract in a mixture of 4 ml of ether-chloroform (1 : 1 V/V). The resulting solution was treated with 4 ml of 10% (W/V) sodium hydroxide. The appearance of a red color indicates the presence of quinones.

Tannins were analyzed by boiling 0.01 g of the extract in 5 ml of water for 5 minutes. After cooling, 5 ml of 2% NaCl (W/V) and 5 ml of 1% gelatin (W/V) were added to the preparation. The appearance of a precipitate characterizes the presence of tannins.

Anthocyanins were detected by boiling 0.01 g of the extract in 5 ml of an aqueous solution of HCl (1% V/V). According to this protocol, the presence of anthocyanins is marked by the appearance of an orange coloration.

The determination of the content of phenols, flavonoids, and tannins in the aqueous extract of *A. ampeloprasum* was performed according to the methods described by Ramde-Tiendrebeogo et al. [39], Chang et al. [40], and Govindappa et al. [41], respectively.

The total phenolic content of the aqueous extract of *A. ampeloprasum* was determined by the modified Folin–Ciocalteu method as Ramde-Tiendrebeogo et al. [39] described. Thus, 100 *μ*l of the Folin–Ciocalteu reagent dissolved in water 10 times and 80 *μ*l of a 20% sodium carbonate reagent were added to 20 *μ*l of the aqueous extract of *A. ampeloprasum* (2 mg/ml). The mixture was shaken and incubated in a water bath at 20°C for 30 minutes. The absorbance was read at 765 nm. The total phenolic content was expressed in milligrams of gallic acid equivalent/gram of extract (mgGAE/gE).

The total flavonoid content was determined using the aluminum chloride colorimetric method described by Chang et al. [40]. Thus, 100 *μ*l of the aqueous extract of *A. ampeloprasum* (2 mg/ml) was mixed with 50 *μ*l of aluminum chloride (1.2%) and 50 *μ*l of potassium acetate (120 mM). The mixture was incubated at room temperature for 30 minutes and the absorbance was read at 415 nm. Total flavonoid content was expressed in milligrams of quercetin equivalent/gram of extract (mgQE/gE).

The total tannin content was estimated using the Folin–Ciocalteu method described by Govindappa et al. [41]. Thus, 100 *μ*l of the aqueous extract of *A. ampeloprasum* (2 mg/ml) was mixed with 500 *μ*l of the Folin–Ciocalteu reagent dissolved in water 10 times, 1000 *μ*l of a 35% sodium carbonate reagent, and 8.4 ml of distilled water. The mixture was shaken and incubated at room temperature for 30 minutes and the absorbance was read at 700 nm. The total tannin content was expressed in milligrams of tannic acid equivalent/gram of the extract (mgTAE/gE).

### 2.3. Dose Determination

The doses of the aqueous extract of *A. ampeloprasum* used in this work were extrapolated from the dosage of Edouard et al. [29] against female infertility. These authors recommend drinking 3 glasses of the decoction of *A. ampeloprasum* daily, during the menstrual period. Considering a glass with a capacity of 250 ml, 3 glasses of the decoction of *A. ampeloprasum*, equivalent to a volume of 750 ml of that decoction, are therefore to be taken daily. After freeze-drying 750 ml of the decoction of *A. ampeloprasum*, a mass of 3.716 g of the aqueous extract of *A. ampeloprasum* was obtained. This mass was divided by 60 (average weight of a woman in kg [42]) to obtain the daily dose, which in this case was 61.93 mg/kg. To obtain the equivalent dose in rats, the human dose (61.93 mg/kg) was multiplied by 6.2 following the recommendations of Nair and Jacob [42]. This method gave an equivalent dose of 384 mg/kg. This dose was divided and multiplied by 2 to obtain the minimum and maximum doses of 192 mg/kg and 768 mg/kg, respectively.

### 2.4. Animals

Healthy young female *Wistar* rats 10–12 weeks old, with an average body weight of 150 g before the experiment, were obtained from the breeding facility of the Research Unit of Animal Physiology and Phytopharmacology (University of Dschang, Cameroon). They were housed in clean plastic cages at room temperature and lit by natural light. All rats had free access to a diet (a standard soy-free rat diet to eliminate exposure to exogenous estrogenic compounds) and tap water *ad libitum*.

### 2.5. Ethical Statement

Animal handling and all experiments were carried out after approval of the research proposal by the Scientific Committee of the Department of Animal Biology of the University of Dschang (Cameroon) on March 01, 2021, in conformity with the EU Directive 2010/63/EU for animal experiments.

### 2.6. Experimental Protocol

#### 2.6.1. PCOS Induction

PCOS was induced with letrozole as we described previously [28]. Briefly, 55 female rats were force-fed with letrozole (a reversible aromatase inhibitor) at a dose of 1 mg/kg for 21 consecutive days. Control animals (*n* = 11) received distilled water instead during this period. The onset of PCOS was confirmed by being overweight and by the blockage of the estrous cycle in the diestrus phase, as we reported previously [27, 28].

#### 2.6.2. Grouping and Treatment of Animals

**Figure 1:**
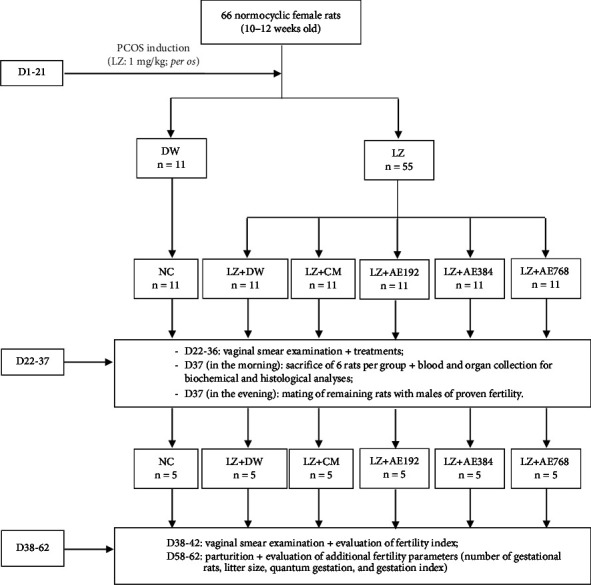
Schematic representation of the experimental protocol. NC: normal control; LZ + DW: negative control; LZ + CM: positive control; AE: aqueous extract of *A. ampeloprasum*; CM: clomiphene citrate + metformin; D: day; DW: distilled water; LZ: letrozole; PCOS: polycystic ovary syndrome.

On the day following the last administration of letrozole or distilled water (on day 22), animals were assigned to the following treatment groups: (i) NC: normal control group composed of healthy animals receiving distilled water during treatment; (ii) LZ + DW: negative control group composed of PCOS animals receiving distilled water during treatment; (iii) LZ + CM: positive control group composed of PCOS animals co-treated with clomiphene citrate (1 mg/kg) and metformin (200 mg/kg); and (iv) LZ + AE: group composed of PCOS animals treated with the aqueous extract of *A. ampeloprasum* at the doses of 192 mg/kg (LZ + AE192), 384 mg/kg (LZ + AE384), and 768 mg/kg (LZ + AE768). Each group consisted of 11 female rats. Treatments were administered orally for 15 consecutive days. Animals were weighed weekly during the experiment (Figure 1).

#### 2.6.3. Estrous Cycle Monitoring

The estrous cycle was examined by daily observation of vaginal smears under the microscope, for 25 days before the induction of PCOS and then, 7 days before the treatments, and throughout the treatment period, as we reported previously [27, 28].

#### 2.6.4. Sacrifice and Collection of Blood and Tissues

At the end of the treatment period, 6 rats per group were sacrificed under anesthesia (diazepam (10 mg/kg) and ketamine (50 mg/kg) administered intraperitoneally). Blood, collected (in dry tubes) from each rat by catheterization of the abdominal artery, was centrifuged at 3000 rpm at room temperature for 15 minutes. The serum obtained was stored at −20°C for biochemical analyses. Abdominal fat, ovaries, and uterus were collected and weighed. The left ovary of each rat was homogenated in 0.9% NaCl (10% W/V) and then centrifuged at 3000 rpm at room temperature for 15 minutes. The supernatant was collected and stored at −20°C for biochemical analyses. The right ovary and uterus were fixed in 10% formalin for histological analysis.

### 2.7. Biochemical Analyses

#### 2.7.1. Measurement of Serum Lipid Levels

Serum levels of triglycerides (TG), total cholesterol (TC), and HDL-cholesterol (HDL-C) were measured by a fully automated enzymatic method using reagent kits purchased from SIGMA Diagnostics (Budapest, Hungary). Atherogenic indexes were determined using the formulae described by Anthony et al. [43] (CT/HDL-C) and Dobiášová and Frohlich [44] (Log (TG)/HDL-C).

#### 2.7.2. Measurement of Serum Hormone Levels

The hormonal profile was examined by measuring serum LH, testosterone, and estradiol levels. Serum levels of these hormones were assessed by ELISA tests using reagent kits purchased from Calbiotech (El Cajon, California, USA). The absorbance of calibrators and the specimen was determined using the ELISA microplate reader, the Multiskan Ascent plate reader, purchased from MTX Lab Systems, Inc. (Bradenton, USA). Hormone concentrations were evaluated by using calibration curves established by the calibrators supplied with the kits.

#### 2.7.3. Measurement of Oxidative Stress-Related Parameters

The levels of malondialdehyde (MDA) in the ovary homogenates were assessed using the method of Wilbur et al. [45]. The principle of this assay stipulates that MDA or MDA-like substances and thiobarbituric acid react at 90–100°C with the production of a pink pigment having an absorption maximum at 532 nm. The ovarian levels of MDA were determined using the following formula:

(1) $$\begin{array}{c} \left[\text{MDA}\right]=10^{9}\times \left(\frac{DO}{\varepsilon .l.m.}\right), \end{array}$$

where [MDA] = concentration of MDA (nM/mg of tissue); DO = absorbance of the sample–absorbance of the reaction blank; *ε* = molar extinction coefficient (1.56 × 10^5^ M^−1^ cm^−1^); *l* = path length (1 cm); and *m* = mass of the ovary used for the preparation of homogenates (mg).

The levels of proteins in the ovary homogenates were assessed using a reagent kit purchased from Randox (London, UK) and following the manufacturer's instructions. This parameter was used to assess ovary levels of antioxidant enzymes (superoxide dismutase, catalase, and total peroxidases) as the amount of each of these enzymes in the ovary homogenates was assessed relative to the total protein content in this tissue.

The measurement of superoxide dismutase (SOD) activity is based on the ability of SOD to inhibit the auto-oxidation of adrenaline to adrenochrome in an alkaline medium. SOD activity in the ovaries was assessed according to the method described by Habbu et al. [46] and the percentage of adrenaline inhibition was calculated as follows:

(2) $$\begin{array}{c} \%I=100\times \left[100-\left(\frac{{\text{DO}}_{\text{sample}}}{{\text{DO}}_{\text{blank}}}\right)\right], \end{array}$$

where %*I* = percentage inhibition of adrenaline oxidation; DO_sample_: average between the absorbance at 30 seconds and the absorbance at 90 seconds of the sample; and DO_blank_: absorbance of the reaction blank.

Considering that 50% inhibition corresponds to one unit, the activity of SOD was expressed in units per amount of proteins as follows:

(3) $$\begin{array}{c} A=\frac{\%I}{\left(50\times p\right)}, \end{array}$$

where *A* = activity of SOD (in unit/mg of total proteins) and *p* = ovarian total protein levels (mg/dl).

Catalase activity in the ovaries was estimated by the method described by Habbu et al. [46]. The measurement of the activity of this enzyme is based on the decomposition of H_2_O_2_ into water by catalase (present in the sample). The concentration of undecomposed H_2_O_2_ was evaluated using a calibration curve established from a standard solution (50 mM H_2_O_2_). Ovary catalase activity was determined as follows:

(4) $$\begin{array}{c} A=\frac{\text{DO}}{a.t.p}, \end{array}$$

where *A* = catalase activity (mole of H_2_O_2_/min/mg of total proteins); DO = absorbance of the sample–absorbance of the reagent blank; *a* = slope of the calibration curve; *t* = reaction time (1 min); and *p* = ovarian total protein level (mg/dl).

Measurement of ovarian levels of total peroxidases was carried out following the method described by Habbu et al. [46]. Briefly, 1 ml of KI solution (10 mM) and 1 ml of sodium acetate (40 mM) were added to 0.5 ml of the ovarian homogenates. After mixing, the absorbance of potassium iodide was read at 353 nm. Then, 20 *μ*l of H_2_O_2_ (15 mM) was added and the change in the absorbance in 5 min was recorded. The amount of total peroxidases in the ovaries was deduced by the law of Beer–Lambert [47] as follows:

(5) $$\begin{array}{c} C=1000\times \left[\frac{\text{DO}}{\varepsilon .l.p.}\right], \end{array}$$

where *C* = concentration of ovarian total peroxidases (mM/mg of total proteins); DO = optical density; *ε* = molar extinction coefficient (11.3 M^−1^ cm^−1^); *l* = path length (1 cm); and *p* = ovarian total protein level (mg/dl).

### 2.8. Fertility Test

After sacrifice, the five remaining rats in each group were mated with males of proven fertility for 5 consecutive days, the average duration of an estrous cycle [27, 28]. The daily (7:30 a.m. to 8:30 a.m.) examination of vaginal smears allowed for determining the fertility index and the first day of gestation. The latter was fixed on the day the spermatozoa were observed in the vaginal smears (positive vaginal smears) as we reported previously [27, 28]. Rats were then followed up until parturition. After parturition, additional parameters of fertility were determined using the formulae we described previously [27].

- Fertility index: 100 × (number of vaginal smear-positive rats/number of mated rats)
- Quantum gestation: 100 × (number of gestational rats/number of vaginal smear-positive rats)
- Gestation index: 100 × (number of rats with viable fetuses at birth/total number of gestational rats)
- Average litter size: total number of pups/number of gestational rats

### 2.9. Histological Analyses

The histological analyses of the ovaries and uterus were performed on 5 *μ*m sections of paraffin-embedded tissues. These sections have been stained with hematoxylin and eosin. Histomorphological changes in these tissues were assessed on photomicrographs using a Scientico STM-50 microscope. The latter was equipped with a Celestron MA411101 camera connected to a computer where the image was transferred and analyzed with Image J1.3 software.

### 2.10. Statistical Analysis

Data are presented as mean ± standard error of the mean (SEM), except for the estrous cycle data which instead show the most represented stage of the estrous cycle in each group as we reported previously [27, 28]. GraphPad Prism 5.03 software was used to perform data analysis. Data from the control and treated groups were compared using a one-way analysis of variance (ANOVA) followed by Tukey's post-test for multiple comparisons. Differences were considered significant at *p* < 0.05.

## 3. Results

### 3.1. Chemical Composition of the Aqueous Extract of *A. ampeloprasum*

Seven classes of compounds were detected in the aqueous extract of *A. ampeloprasum*. These include phenols, flavonoids, triterpenoids, tannins, saponins, quinones, and anthocyanins (Table 1).

Quantitatively, the amounts of *A. ampeloprasum* in phenols, flavonoids, and tannins were 4.58 ± 0.23 mgGAE/gE, 1.40 ± 0.33 mgQE/gE, and 1.48 ± 0.31 mgTAE/gE, respectively (Table 2).

### 3.2. Effects of the Aqueous Extract of *A. ampeloprasum* on the Body Weight

**Figure 2:**
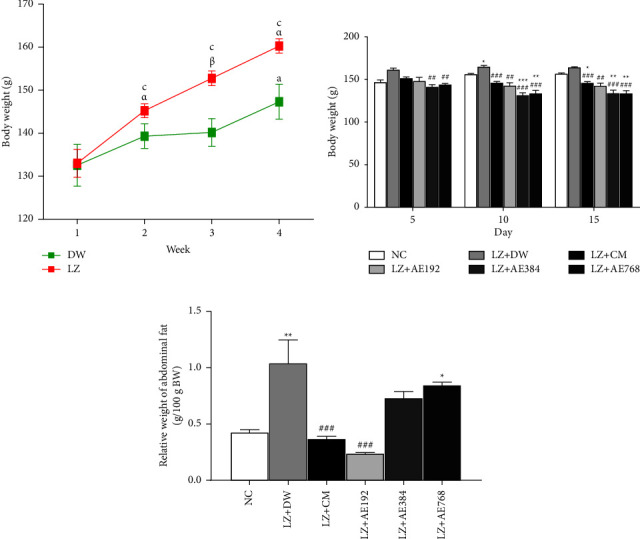
Weekly change of animal body weight during the induction of PCOS (a) and effects of the aqueous extract of *A. ampeloprasum* on the body weight (b) and the relative weight of abdominal fat (c). NC: normal control; LZ + DW: negative control; LZ + CM: positive control; AE: aqueous extract of *A. ampeloprasum*; CM: clomiphene citrate + metformin; DW: distilled water; LZ: letrozole. Results are presented as mean ± S.E.M. n = 6; ^*α*^*p* < 0.05 and ^*β*^*p* < 0.01 vs DW; ^a^*p* < 0.05 and ^c^*p* < 0.001 vs week 1; ^*∗*^*p* < 0.05, ^*∗∗*^*p* < 0.01 and ^*∗∗∗*^*p* < 0.001 vs NC; ^##^*p* < 0.01 and ^###^*p* < 0.001 vs LZ + DW.

At the beginning of the experiment (week 1), the body weight of animals to be used as control and that of animals intended to receive letrozole (1 mg/kg) were comparable: 132.54 ± 4.88 g and 133.02 ± 3.21 g, respectively. During the experiment, a gradual increase in the body weight was observed in both groups. In the control group, the body weight was 132.54 ± 4.88 g at week 1. This parameter increased to 139.33 ± 2.89 g at week 2 (5% increase), 140.2 ± 3.22 g (6% increase) at week 3 and 147.30 ± 4.06 g (11% increase; *p* < 0.05) at week 4. In animals receiving letrozole, the body weight increased from 133.02 ± 3.21 g at week 1 to 145.22 ± 1.63 g (9% increase; *p* < 0.001) at week 2, 152 .77 ± 1.72 g (15% increase; *p* < 0.001) at week 3, and 160.25 ± 1.66 g (21% increase; *p* < 0.001) at week 4. In comparison with the control group, the body weight of animals receiving letrozole increased by 4% (*p* < 0.05) after one week of letrozole administration and 9% after two (*p* < 0.01) and three (*p* < 0.05) weeks of letrozole administration (Figure 2(a)).

During the 15 days of treatment, the body weight of the negative control remained higher than that of the normal control. Clomiphene citrate and metformin reduced animal body weight by 6%, 12% (*p* < 0.001), and 11% (*p* < 0.01) after 5, 10, and 15 days of treatment, respectively, compared to the negative control. The same observation was made in animals treated with the aqueous extract of *A. ampeloprasum* at the doses tested, in comparison with the negative control (Figure 2(b)):

- At 192 mg/kg, *A. ampeloprasum* reduced body weight by 8%, 14%, and 13% after 5, 10, and 15 days of treatment, respectively;
- At 384 mg/kg, *A. ampeloprasum* reduced body weight by 12% (*p* < 0.001), 21% (*p* < 0.001), and 18% (*p* < 0.001) after 5, 10, and 15 days of treatment, respectively;
- At 768 mg/kg, *A. ampeloprasum* reduced body weight by 11% (*p* < 0.01) after 5 days of treatment and 19% (*p* < 0.001) after 10 and 15 days of treatment, respectively.

Figure 2(c) shows that the relative weight of abdominal fat increased by 145% (*p* < 0.01) in the negative control group where the abdominal fat weighed 1.035 ± 0.214 g/100 g BW, in comparison with the normal control where this parameter was on average 0.423 ± 0.031 g/100 g BW. The combined administration of clomiphene citrate and metformin reduced the relative weight of abdominal fat by 65% (*p* < 0.001) as compared to the negative control. A similar effect was observed with the aqueous extract of *A. ampeloprasum* which reduced the relative weight of abdominal fat by 77% (*p* < 0.001) at the dose of 192 mg/kg, 30% at the dose of 384 mg/kg, and 19% at the dose of 768 mg/kg.

### 3.3. Effects of the Aqueous Extract of *A. ampeloprasum* on Lipidemia

**Figure 3:**
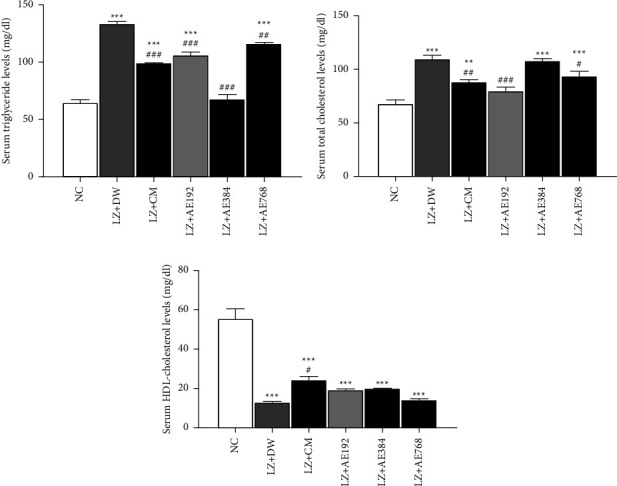
Effects of the aqueous extract of *A. ampeloprasum* on serum levels of triglycerides (a), total cholesterol (b), and HDL-cholesterol (c). NC: normal control; LZ + DW: negative control; LZ + CM: positive control; AE: aqueous extract of *A. ampeloprasum*; CM: clomiphene citrate + metformin; DW: distilled water; LZ: letrozole. Results are presented as mean ± S.E.M., *n* = 6, ^*∗∗*^*p* < 0.01 and ^*∗∗∗*^*p* < 0.001 vs NC, ^#^*p* < 0.05, ^##^*p* < 0.01 and ^###^*p* < 0.001 vs LZ + DW.

Serum triglyceride (TG) levels increased by 106% (*p* < 0.001) in the negative control as compared to those of the normal control. The combined administration of clomiphene citrate and metformin decreased this parameter by 26% (*p* < 0.001) in comparison with the negative control. A similar effect (decrease in serum TG levels) was observed with the aqueous extract of *A. ampeloprasum* at the doses tested: 20% decrease at 192 mg/kg (*p* < 0.001), 49% decrease at 384 mg/kg (*p* < 0.001), and 13% decrease at 768 mg/kg (*p* < 0.01) (Figure 3(a)).

Figure 3(b) shows that serum total cholesterol levels increased by 63% (*p* < 0.001) in the negative control relative to those of the normal control. The co-treatment with clomiphene citrate and metformin reduced this parameter by 20% (*p* < 0.01) as compared to the negative control. *A. ampeloprasum* induced a similar effect by decreasing serum total cholesterol levels at the doses of 192 mg/kg (27% decrease; *p* < 0.001) and 768 mg/kg (15% decrease; *p* < 0.05), in comparison with the negative control.

Serum HDL cholesterol levels decreased by 77% (*p* < 0.001) in the negative control relative to those of the normal control. The co-treatment with clomiphene citrate and metformin increased this parameter by 90% (*p* < 0.05) as compared to the negative control. A similar increase in serum HDL-cholesterol levels was observed with *A. ampeloprasum* at the doses tested: a 50% increase at 192 mg/kg, 56% increase at 384 mg/kg, and an 11% increase at 768 mg/kg, in comparison with the negative control (Figure 3(c)).

**Figure 4:**
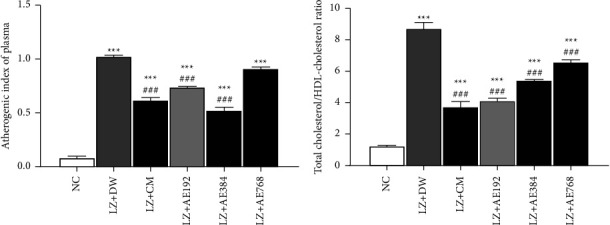
Effects of the aqueous extract of *A. ampeloprasum* on the atherogenic index of plasma (a) and the total cholesterol/HDL-cholesterol ratio (b). NC: normal control; LZ + DW: negative control; LZ + CM: positive control; AE: aqueous extract of *A. ampeloprasum*; CM: clomiphene citrate + metformin; DW: distilled water; LZ: letrozole. Results are presented as mean ± S.E.M., *n* = 6, ^*∗∗∗*^*p* < 0.001 vs NC, ^###^*p* < 0.001 vs LZ + DW.

The atherogenic index of plasma (AIP) was 0.086 ± 0.021 in the normal control versus 1.020 ± 0.027 in the negative control, an increase of 1092% (*p* < 0.001). After co-treatment with clomiphene citrate and metformin, the AIP value decreased by 40% (*p* < 0.001) in comparison with the negative control. The aqueous extract of *A. ampeloprasum* induced a similar effect (decrease in the AIP value) at the doses tested: 27% decrease at 192 mg/kg (*p* < 0.001), 49% decrease at 384 mg/kg (*p* < 0.001), and 11% decrease at 768 mg/kg, in comparison with the negative control (Figure 4(a)).

The total-cholesterol/HDL-cholesterol (TC/HDL-C) ratio went from 1.262 ± 0.093 in the normal control to 8.726 ± 0.453 in the negative control, an increase of 592% (*p* < 0.001). The combined administration of clomiphene citrate and metformin decreased this ratio by 57% (*p* < 0.001), in comparison with the negative control. *A. ampeloprasum* also decreased the TC/HDL-C ratio by 52% (*p* < 0.001) at the dose of 192 mg/kg, 38% (*p* < 0.001) at the dose of 384 mg/kg, and 24% (*p* < 0.001) at the dose of 768 mg/kg, in comparison with the negative control (Figure 4(b)).

### 3.4. Effects of the Aqueous Extract of *A. ampeloprasum* on the Course of the Estrous Cycle

**Figure 5:**
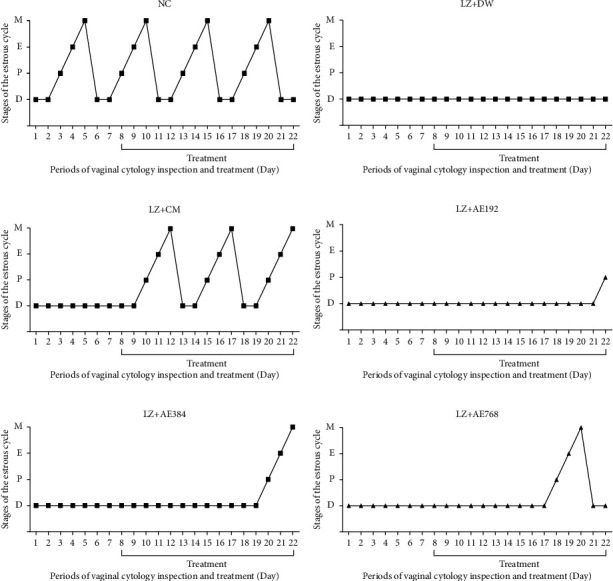
Effects of the aqueous extract of *A. ampeloprasum* on the course of the estrous cycle. NC: normal control (a); LZ + DW: negative control (b); LZ + CM: positive control (c); AE-treated animals (d–f); AE: aqueous extract of *A. ampeloprasum*; CM: clomiphene citrate + metformin; DW: distilled water; LZ: letrozole. Data show the most represented phase of the estrous cycle in each group, *n* = 6, P = proestrus, E = estrus, M = metestrus, and D = diestrus.

The average duration of the estrous cycle in the normal control was 5 days and was made up of four stages including proestrus, estrus, metestrus, and diestrus. While each of the first three phases of the estrous cycle varied over 24 hours, the last phase (the diestrus) spanned two days (Figure 5(a)). In the negative control, the estrous cycle was blocked in the diestrus phase throughout the experiment (Figure 5(b)). In animals co-treated with clomiphene citrate and metformin, the cyclic occurrence of the different phases of the estrous cycle from the proestrus phase, resumed after two days of treatment (Figure 5(c)). With *A. ampeloprasum*, the proestrus appeared after 14 days of treatment at the dose of 192 mg/kg (Figure 5(d)), 12 days of treatment at the dose of 384 mg/kg (Figure 5(e)), and 10 days of treatment at the dose of 768 mg/kg (Figure 5(f)).

### 3.5. Effects of the Aqueous Extract of *A. ampeloprasum* on the Hormonal Profile

**Figure 6:**
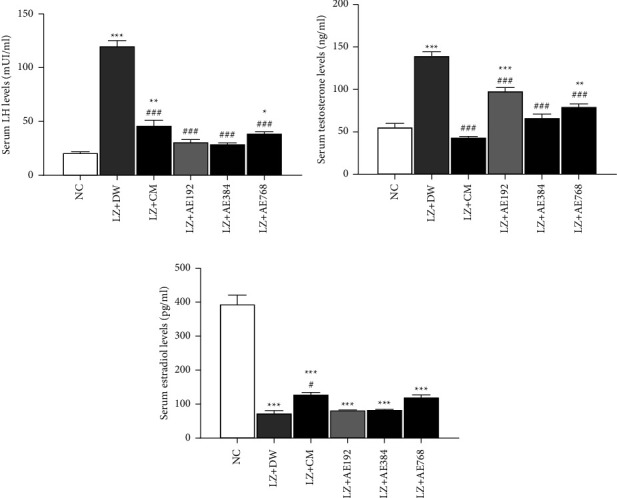
Effects of the aqueous extract of *A. ampeloprasum* on serum levels of LH (a), testosterone (b), and estradiol (c). NC: normal control; LZ + DW: negative control; LZ + CM: positive control; AE: aqueous extract of *A. ampeloprasum*; CM: clomiphene citrate + metformin; DW: distilled water; LZ: letrozole. Results are presented as mean ± S.E.M., *n* = 6, ^*∗*^*p* < 0.05, ^*∗∗*^*p* < 0.01 and ^*∗∗∗*^*p* < 0.001 vs NC, and ^#^*p* < 0.05 and ^###^*p* < 0.001 vs LZ + DW.

Serum LH levels went from 21.474 ± 1.741 mIU/ml in the normal control to 120.522 ± 6.087 mIU/ml in the negative control, an increase of 461% (*p* < 0.001). This parameter decreased by 61% (*p* < 0.001) after the combined administration of clomiphene citrate and metformin, in comparison with the negative control. A similar decrease in serum LH levels was observed with the aqueous extract of *A. ampeloprasum* at the doses tested: 74% decrease at the dose of 192 mg/kg (*p* < 0.001), 76% decrease at the dose of 384 mg/kg (*p* < 0.001), and 67% decrease at the dose of 768 mg/kg (*p* < 0.001), in comparison with the negative control (Figure 6(a)).

Serum testosterone levels went from 56.256 ± 4.926 ng/ml in the normal control to 140.225 ± 5.012 ng/ml in the negative control, an increase of 149% (*p* < 0.001). Clomiphene citrate and metformin lowered the mean value of serum testosterone levels by 69% (*p* < 0.001) in comparison with the negative control. A similar effect (decrease in serum testosterone levels) was induced by the aqueous extract of *A. ampeloprasum* at the doses tested: 30% decrease at the dose of 192 mg/kg (*p* < 0.001), 52% decrease at the dose of 384 mg/kg (*p* < 0.001), and 43% decrease at the dose of 768 mg/kg (*p* < 0.001), in comparison with the negative control (Figure 6(b)).

Serum estradiol levels decreased from 395.452 ± 25.498 pg/ml in the normal control to 75.030 ± 6.927 pg/ml in the negative control, a decrease of 81% (*p* < 0.001). This parameter increased by 74% (*p* < 0.05) after the combined administration of clomiphene citrate and metformin, in comparison with the negative control. Following treatment with *A. ampeloprasum*, serum estradiol levels did not change statistically compared to the negative control, although a slight increase in this parameter was observed at the doses tested: an increase of 12% at 192 mg/kg, 13% increase at 384 mg/kg, and 62% increase at 768 mg/kg (Figure 6(c)).

### 3.6. Effects of the Aqueous Extract of *A. ampeloprasum* on the Relative Weight of the Ovaries and Uterus and the Uterine Epithelial Height

**Figure 7:**
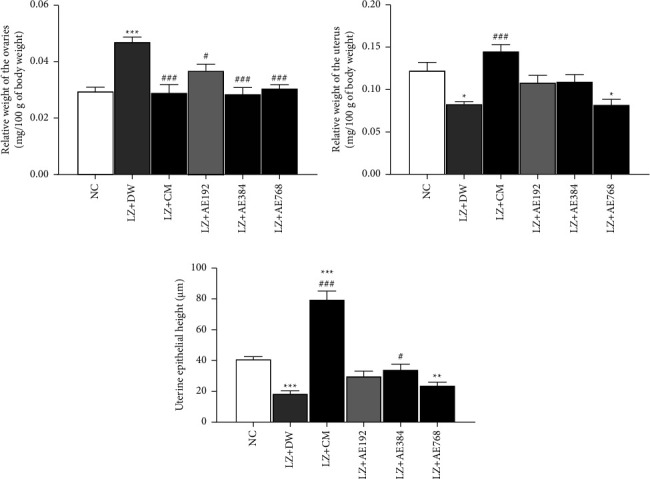
Effect of the aqueous extract of *A. ampeloprasum* on the relative weights of the ovaries (a), uterus (b), and the uterine epithelial height (c). NC: normal control; LZ + DW: negative control; LZ + CM: positive control; AE: aqueous extract of *A. ampeloprasum*; CM: clomiphene citrate + metformin; DW: distilled water; LZ: letrozole. Results are presented as mean ± S.E.M., *n* = 6, ^*∗*^*p* < 0.05, ^*∗∗*^*p* < 0.01 and ^*∗∗∗*^*p* < 0.001 vs NC, and ^#^*p* < 0.05 and ^###^*p* < 0.001 vs LZ + DW.

Figure 7(a) shows that the relative weight of the ovaries increased by 59% (*p* < 0.001) in the negative control, relative to the normal control. After co-treatment with clomiphene citrate and metformin, the relative weight of the ovaries decreased by 38% (*p* < 0.001), in comparison with the negative control. A similar effect was induced by the aqueous extract of *A. ampeloprasum* as it decreased the relative weight of the ovaries by 21% (*p* < 0.05) at the dose of 192 mg/kg, 39% (*p* < 0.001) at the dose of 384 mg/kg, and 35% (*p* < 0.001) at the dose of 768 mg/kg, as compared to the negative control.

The graphic representation of the relative weight of the uterus shows that this parameter decreased by 32% (*p* < 0.05) in the negative control, as compared to the normal control. After co-treatment with clomiphene citrate and metformin, the relative weight of the uterus increased by 75% (*p* < 0.001), in comparison with the negative control. A similar increase in the relative uterine weight was observed with the aqueous extract of *A. ampeloprasum* at the doses of 192 mg/kg (30% increase) and 384 mg/kg (32% increase), in comparison with the negative control (Figure 7(b)).

The uterine epithelial height decreased by 54% (*p* < 0.001) in the negative control, as compared to the normal control. The combined administration of clomiphene citrate and metformin increased this parameter by 323% (*p* < 0.001), in comparison with the negative control. The aqueous extract of *A. ampeloprasum* induced a similar effect (increase in the uterine epithelial height) at the doses tested: 60% increase at the dose of 192 mg/kg, 82% increase at the dose of 384 mg/kg (*p* < 0.05), and 28% increase at the dose of 768 mg/kg, in comparison with the negative control (Figure 7(c)).

### 3.7. Effects of the Aqueous Extract of *A. ampeloprasum* on the Ovarian Follicle Growth

**Figure 8:**
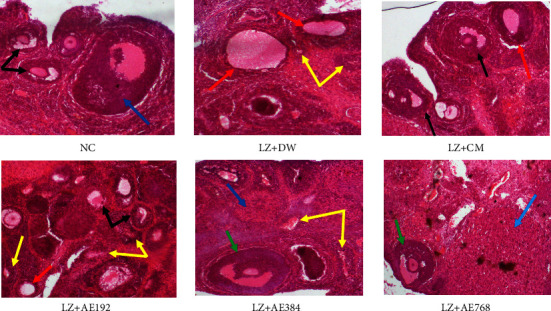
Photomicrographs (X 200, hematoxylin and eosin staining) of the ovaries of experimental animals. NC: normal control; LZ + DW: negative control; LZ + CM: positive control; AE: aqueous extract of *A. ampeloprasum*; CM: clomiphene citrate + metformin; DW: distilled water; LZ: letrozole. Black arrow: tertiary follicle; green arrow: Graafian follicle; red arrow: cystic follicle; yellow arrow: atretic follicle; and blue arrow: *corpora lutea*.

The growth of ovarian follicles was analyzed on photomicrographs of ovarian sections (Figure 8). The analysis was performed by counting the different types of ovarian follicles. These include primary, secondary, tertiary, and Graafian follicles, *corpora lutea*, and cystic and atretic follicles. In the negative control, the number of primary, secondary, tertiary and Graafian follicles and *corpora lutea* decreased by at least 39%, as compared to the normal control. In contrast, the number of cystic and atretic follicles increased by at least 80% in the negative control, relative to the normal control. Clomiphene citrate and metformin reversed the effects observed in the negative control as they increased (at least 53% increase) the number of primary, secondary, tertiary, and Graafian follicles and *corpora lutea*. Cystic and atretic follicles decreased by 73% (*p* < 0.001), as compared to the negative control. *A. ampeloprasum* induced effects similar to those induced by clomiphene citrate and metformin at the doses tested (Table 3).

### 3.8. Effects of the Aqueous Extract of *A. ampeloprasum* on Oxidative Stress-Related Parameters in the Ovaries

**Figure 9:**
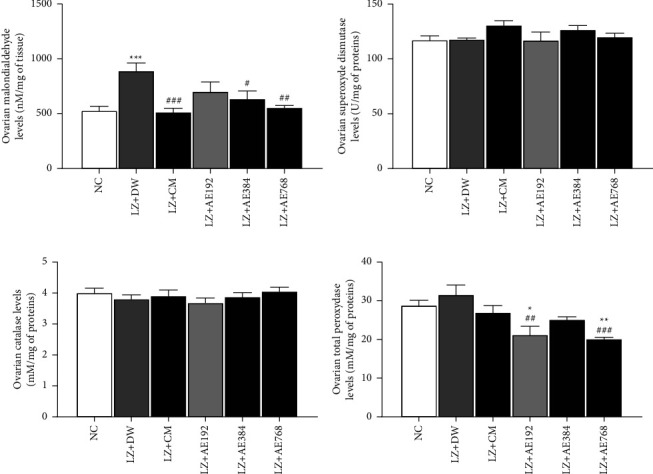
Effects of the aqueous extract of *A. ampeloprasum* on the ovarian levels of malondialdehyde (a), superoxide dismutase (b), catalase (c), and total peroxidases (d), NC: normal control; LZ + DW: negative control; LZ + CM: positive control; AE: aqueous extract of *A. ampeloprasum*; CM: clomiphene citrate + metformin; DW: distilled water; LZ: letrozole. Results are presented as mean ± S.E.M. *n* = 6, ^*∗*^*p* < 0.05, ^*∗∗*^*p* < 0.01 and ^*∗∗∗*^*p* < 0.001 vs NC; ^#^*p* < 0.05, ^##^*p* < 0.01 and ^###^*p* < 0.001 vs LZ + DW.

The ovarian levels of malondialdehyde (MDA) was 532.051 ± 35.805 nM/mg of tissues in the normal control versus 895.299 ± 67.034 nM/mg of tissues in the negative control, an increase of 68% (*p* < 0.001). The co-treatment with clomiphene citrate and metformin decreased ovarian MDA levels by 42% (*p* < 0.001), as compared to the negative control. A similar decrease in this parameter was observed with the aqueous extract of *A. ampeloprasum* at the doses tested: 21% decrease at the dose of 192 mg/kg, 28% decrease at the dose of 384 mg/kg (*p* < 0.05), and 38% decrease at the dose of 768 mg/kg (*p* < 0.01), in comparison with the negative control (Figure 9(a)).

Concerning the ovarian superoxide dismutase (SOD) level, results show that this parameter did not vary significantly between the normal control (117.670 ± 3.445 U/mg of proteins) and the negative control (118.164 ± 1.421 U/mg of proteins). The different treatments administered (clomiphene citrate and metformin, and the aqueous extract of *A. ampeloprasum*) did not significantly affect ovarian SOD level which remained close to that of the negative control (Figure 9(b)).

Results on the ovarian catalase level show that this parameter did not vary significantly between the normal control (4.020 ± 0.137 mM/mg of proteins) and the negative control (3.818 ± 0.126 mM/mg of proteins). Clomiphene citrate and metformin, as well as the aqueous extract of *A. ampeloprasum*, did not significantly affect the ovarian catalase level which remained close to that of the negative control (Figure 9(c)).

Ovarian total peroxidase levels were 28.925 ± 1.195 mM/mg of proteins in the normal control versus 31.638 ± 2.434 mM/mg of proteins in the negative control, an increase of 9%. The combined administration of clomiphene citrate and metformin reduced this parameter by 15%, in comparison with the negative control. The aqueous extract of *A. ampeloprasum* induced a similar effect by decreasing ovarian total peroxidase levels by 33% (*p* < 0.01) at the dose of 192 mg/kg, 20% at the dose of 384 mg/kg, and 36% (*p* < 0.001) at the dose of 768 mg/kg, as compared to the negative control (Figure 9(d)).

### 3.9. Effects of the Aqueous Extract of *A. ampeloprasum* on the Fertility of Rats with PCOS

The fertility index was 80% in the normal control versus 0% in the negative control. After the combined administration of clomiphene citrate and metformin, this index increased to 100%. The aqueous extract of *A. ameploprasum* induced a similar effect (increase in the fertility index) at the doses tested: 60% increase at the dose of 192 mg/kg, and 80% increase at the doses of 384 and 768 mg/kg (Table 4).

The quantum gestation as well as the gestation index was 100% in the normal control versus 0% in the negative control. The co-treatment with clomiphene citrate and metformin, as well as the aqueous extract of *A. ampeloprasum*, increased each of these parameters to 100% (Table 4).

The total number of pups per group was 0 in the negative control versus 35 in the normal control. After the co-treatment with clomiphene citrate and metformin, the total number of pups was 34. The aqueous extract of *A. ampeloprasum* also increased this parameter at the doses tested: 18 pups at the dose of 192 mg/kg, 30 pups at the dose of 384 mg/kg, and 25 pups at the dose of 768 mg/kg (Table 4).

The average litter size was 8.75 pups per female in the normal control versus 0 pups per female in the negative control. The co-treatment with clomiphene citrate and metformin raised this parameter to a value of 6.8 pups per female. A similar observation was made with the aqueous extract of *A. ampeloprasum* where the average litter size was 6 pups per female at the dose of 192 mg/kg, 7.5 pups per female at the dose of 384 mg/kg, and 6.25 pups per female at the dose of 768 mg/kg (Table 4).

## 4. Discussion

The present study aimed to evaluate the effects of the aqueous extract of *A. ampeloprasum* on some metabolic and reproductive disorders associated with PCOS in female *Wistar* rats. PCOS was induced with a reversible aromatase inhibitor (letrozole) as we reported previously [28]. Consistent with our previous results [27, 28], the daily administration of the dose of 1 mg/kg of letrozole for 21 consecutive days induced a PCOS rat model possessing similar metabolic (overweight and dyslipidemia) and reproductive (hyperandrogenism, high LH levels, ovarian cysts, and infertility) disorders as seen in PCOS women. To induce this phenotype, letrozole acts by preventing the conversion of androgen to estrogens [48]. Excess androgens induce the excessive release of LH by the pituitary gland [24] and promote the ovarian production of androgens, weight gain, anovulation, and cyst formation in the ovaries, either directly or through a mechanism of action involving insulin [11–18, 49]. Insulin was also found to inhibit the activity of adenosine monophosphate-activated protein kinase (AMPK) [50]. Low AMPK activity promotes lipogenesis (biosynthesis of fatty acids) [51] and hypercholesterolemia [52].

*A. ampeloprasum* reversed letrozole-induced effects as it decreased animal body weight and abdominal fat accumulation. This effect was associated with decreased serum testosterone levels. Indeed, androgen is known to induce visceral obesity by stimulating the differentiation of pre-adipocytes into adipocytes preferentially in the abdomen [49]. Therefore, by decreasing testosterone levels, *A. ampeloprasum* inhibited lipogenesis signaling pathways (inhibition of AMPK and differentiation of pre-adipocytes into adipocytes), thereby reducing fat accumulation and consequently body weight. The significant difference between control animals and animals treated with *A. ampeloprasum* in terms of body weight may be the result of an inhibition of weight gain, while animals in the control groups continued to grow.

Also, since hyperandrogenism indirectly inhibits AMPK activity by stimulating hyperinsulinemia [11, 50], the effects induced by *A. ampeloprasum* suggest an enhanced AMPK activity, hence the reduction of serum triglyceride levels in *A. ampeloprasum*-treated animals, as triglycerides are synthesized by the esterification of fatty acids to glycerol. Results on the other lipid parameters show that *A. ampeloprasum* decreased serum total cholesterol levels and increased serum HDL-cholesterol levels. The mechanism through which *A. ampeloprasum* induced these effects may include (i) the reduction of the activity of 3-hydroxy-3-methylglutaryl coenzyme A (HMG-CoA) reductase and/or cholesterol breakdown and (ii) the increase in the activity of lecithin: cholesterol acyl transferase (LCAT) and/or the inhibition of the hepatic HDL-cholesterol uptake. HMG-CoA reductase is the rate-limiting enzyme involved in the biosynthesis of cholesterol in the liver [52]. Its activity is inactivated by AMPK as cholesterol synthesis was found to be significantly suppressed in response to AMPK activators [52]. This at least partly supports the hypothesis that *A. ampeloprasum* stimulates AMPK activity, thereby suppressing cholesterol synthesis. Cholesterol breakdown could be attributed to flavonoids present in *A. ampeloprasum*, as this class of compounds has been shown to increase hepatic expression of the cytochrome P450 gene 7A1 [53] which codes for cholesterol-7*α*-hydroxylase, an enzyme that stimulates the conversion of cholesterol into bile acids in the liver [54].

LCAT is a plasma enzyme that esterifies peripheral tissue cholesterol onto nascent pre-*β* HDL (small complexes formed by the association of apolipoprotein A-1 (apoA1) (produced by the liver) with hepatic phospholipids and cholesterol via interaction with the ATP-binding cassette transporter A1 (ABCA1)), thus forming mature (larger) *α*-HDL, the major HDL species found in plasma [55–57]. LCAT deficiency prevents the formation of mature HDL, leading to an overall decrease in HDL levels [55, 57]. Therefore, the increase in serum HDL-cholesterol levels in rats treated with *A. ampeloprasum* indicates that this vegetable (or leek) would have promoted the hepatic production of apoA1 and increased the activities of ABCA1 and LCAT. This effect could be attributed to the flavonoids present in the aqueous extract of *A. ampeloprasum*, because in our previous work, it was shown that isoflavones promote the formation of HDL as they leaned the *Apoa1*/*Scarb1* balance in favor of *Apoa1* [53]. *Apoa1* and *Sacrb1* are estrogen-sensitive genes associated with HDL synthesis (*Apoa1*; a gene coding for apolipoprotein A1) and clearance (*Scarb1*; a gene coding for SRB1, a receptor promoting the rapid clearance of HDL-cholesterol and its transport into bile) [53, 58]. The improvement in lipid metabolism induced by the aqueous extract of *A. ampeloprasum* is consistent with the observations of Rahimi-Madiseh et al. [30] who reported the ability of the hydroalcoholic extract of Iranian leek to correct dyslipidemia in diabetic rats. This improvement in lipid metabolism contributed to reducing the atherogenic risk.

Regarding reproductive disorders associated with PCOS (hyperandrogenism, high serum LH levels, blockage of the estrous cycle in the diestrus phase, anovulation, ovarian cysts, and infertility), our results showed that the aqueous extract of *A. ampeloprasum* stimulated the resumption of the estrous cycle, improved ovarian dynamics, decreased serum LH and testosterone levels, and slightly increased serum estradiol levels. The decrease in serum testosterone levels suggests that *A. ampeloprasum* reversed the inhibitory effect of letrozole on aromatase activity. However, the nonsignificant change in estradiol levels somewhat refutes this hypothesis and suggests that *A. ampeloprasum* decreased LH and testosterone levels through a different pathway than aromatase activation. The following hypothesis could be put forward to try to explain the decrease in LH and testosterone levels: Indeed, by inhibiting aromatase activity, letrozole causes an accumulation of androgens leading to hyperandrogenism. The latter causes hyperinsulinemia [11] which in turn increases glutamate levels in the brain [12]. High levels of this excitatory neurotransmitter overstimulate hypothalamic GnRH neurons. The resulting excess release of GnRH causes the pituitary gland to release LH accordingly [12]. *A. ampeloprasum* could have acted at the central level by promoting glutamate reuptake and therefore reducing its excitatory effects on hypothalamic GnRH neurons, thereby reducing pituitary release of LH and consequently the ovarian production of testosterone. This hypothesis paves the way for further research that will elucidate the mechanism by which *A. ampeloprasum* decreases serum LH and testosterone levels in PCOS rats.

The reduction of serum testosterone levels induced by *A. ampeloprasum* contributed to the restoration of ovarian dynamics characterized by an increase in the number of tertiary and Graafian follicles (an indicator of follicle development and maturation) and *corpora lutea* (an indicator of ovulation), and a decrease in the number of cystic and atretic follicles. Literature reports that granulosa cell apoptosis is responsible for the increased number of atretic follicles in PCOS rats. Granulosa cells undergo apoptosis due to low estradiol levels associated with letrozole-induced aromatase inhibition [59]. Thus, *A. ampeloprasum* would have induced an estrogenic-like effect by preventing granulosa cells from atresia, hence the increase in tertiary and Graafian follicles. This hypothesis of the estrogenic potential of *A. ampeloprasum* is confirmed by the hypertrophy of the uterus of treated animals. It is known that the hypertrophy of this main target of estrogens is mediated by the estrogen receptor alpha [60]. The flavonoids present in this plant could be responsible for its estrogenic effect, as isoflavones are known to be estrogen receptor ligands and therefore estrogen-mimicking compounds [32, 33, 53].

Additionally, cellular apoptosis is known to be initiated by reactive oxygen species (ROS) whose elevated levels indicate oxidative stress [61]. Oxidative stress markers were found to be elevated in the serum and ovaries of animals with PCOS [27, 59, 62, 63]. In agreement with these reports, our results showed elevated levels of ovarian malondialdehyde (MDA, indicator of lipid peroxidation) associated with a slight increase in total peroxidases in the negative control. The latter would have increased to reduce the damage caused by oxidative stress on granulosa cells. Thus, low estradiol levels increased oxidative stress in the ovaries of animals with PCOS. This could be the origin of the increase in the process of follicle atresia observed in the negative control. The aqueous extract of *A. ampeloprasum* reversed this effect by decreasing ovarian MDA levels and increasing the levels of total peroxidases. Indeed, substances with antioxidant properties have been reported to decrease ROS production [62], lipid peroxidation [59, 62, 63], and follicle atresia [59, 63]. These data support the antioxidant properties of *A. ampeloprasum* reported by Rahimi-Madiseh et al. [30] in diabetic rats and could be attributed to anthocyanins found in *A. ampeloprasum*.

These beneficial effects of *A. ampeloprasum* could be attributed to its chemical composition which revealed the presence of phenols, flavonoids, terpenoids, and anthocyanins. These classes of compounds were found to have antioxidant, hypolipidemic, hypoandrogenic, and estrogenic properties [31–33, 35–37]. Additionally, the corrective effects of *A. ampeloprasum* on hormonal profile and ovaries created a favorable physiological environment for fertility. Indeed, our results showed an increase in gestation index, quantum gestation, fertility index, and average litter size in animals treated with *A. ampeloprasum*. This confirms at least in part the traditional use of this plant against female infertility [29].

In conclusion, the aqueous extract of *A. ampeloprasum* improved PCOS-impaired physiological parameters in rats through its hypoandrogenic, hypolipidemic, estrogenic, and antioxidant abilities. The corrective effects of *A. ampeloprasum* on PCOS-related infertility justify the traditional use of this plant for the treatment of female infertility. Also, the overall results indicate that doses ranging from 192 mg/kg to 768 mg/kg are within the therapeutic range of the aqueous extract of *A. ampeloprasum*. Traditional practitioners could therefore prescribe a dose twice less than that currently prescribed to treat female infertility. Finally, in line with the suggestion made by Rahimi-Madiseh et al. [30] who proposed the use of *A. ampeloprasum* as a dietary supplement in diabetic patients, the present work suggests that supplementing PCOS patients with this vegetable may also be helpful as they experience similar metabolic disorders seen in diabetic patients.

## Figures and Tables

**Table 1:** Classes of compounds detected in the aqueous extract of *A. ampeloprasum*.

Classes of compounds	*A. ampeloprasum*
Alkaloids	−
Phenols	+
Flavonoids	+
Sterols	−
Triterpenoids	+
Tannins	+
Saponins	+
Anthocyanins	+
Quinones	+

+: detectable; −: not detectable.

**Table 2:** Phenol, flavonoid, and tannin contents in the aqueous extract of *A. ampeloprasum*.

	Total phenol content (mgAGE/gE)	Total flavonoid content (mgQE/gE)	Total tannin content (mgTAE/gE)
Butylhydroxytoluene	426.85 ± 0.68	61.89 ± 0.97	52.43 ± 0.31
*A. ampeloprasum*	4.58 ± 0.23	1.40 ± 0.33	1.48 ± 0.31

**Table 3:** Effects of the aqueous extract of *A. ampeloprasum* on the number of different types of ovarian follicles.

Groups	Primary follicles	Secondary follicles	Tertiary follicles	Graafian follicles	*Corpora lutea*	Cystic follicles	Atretic follicles
NC	3.33 ± 0.39	2.60 ± 0.32	3.40 ± 0.48	2.60 ± 0.32	10.20 ± 0.65	0.00 ± 0.00	5.00 ± 0.36
LZ + DW	1.40 ± 0.32	1.31 ± 0.30	1.40 ± 0.32^###^	1.60 ± 0.41	5.63 ± 0.47^*∗∗∗*^	10.20 ± 0.47^*∗∗∗*^	9.00 ± 0.25^*∗∗∗*^
LZ + CM	2.60 ± 0.66	3.20 ± 0.30	4.56 ± 0.72	5.00 ± 0.36^*∗*^^,###^	8.60 ± 0.66^#^	2.80 ± 0.54^*∗∗*^^,###^	2.40 ± 0.32^*∗*^^,###^
LZ + AE192	3.00 ± 0.57	3.20 ± 0.40	2.70 ± 0.54	2.60 ± 0.61	6.40 ± 0.75^*∗∗*^	4.00 ± 0.57^*∗∗∗*^^,###^	6.66 ± 0.80^#^
LZ + AE384	3.96 ± 0.50^#^	3.80 ± 0.65^##^	2.40 ± 0.20	3.00 ± 0.57	7.70 ± 0.59	3.80 ± 0.30^*∗∗∗*^^,###^	6.26 ± 0.43^##^
LZ + AE768	5.80 ± 0.54^*∗*^^,###^	3.20 ± 0.60	3.20 ± 0.40	4.20 ± 0.54^##^	8.80 ± 0.70^#^	3.00 ± 0.44^*∗∗∗*^^,###^	5.30 ± 0.60^###^

NC: normal control, LZ + DW: negative control; LZ + CM: positive control; AE: aqueous extract of *A. ampeloprasum*; CM: clomiphene citrate + metformin; DW: distilled water; and LZ: letrozole. Results are presented as mean ± S.E.M. *n* = 6, ^*∗*^*p* < 0.05, ^*∗∗*^*p* < 0.01, and ^*∗∗∗*^*p* < 0.001 vs NC; ^#^*p* < 0.05, ^##^*p* < 0.01, and ^###^*p* < 0.001 vs LZ + DW.

**Table 4:** Effects of aqueous extract of *A. ampeloprasum* on some fertility parameters in rats with PCOS.

Groups	NMR	NPR	FI (%)	NGR	QG (%)	GI (%)	TNP	ALS
NC	5	4	80	4	100	100	35	8.75
LZ + DW	5	0	0	0	0	0	0	0
LZ + CM	5	5	100	5	100	100	34	6.8
LZ + AE192	5	3	60	3	100	100	18	6
LZ + AE384	5	4	80	4	100	100	30	7.5
LZ + AE768	5	4	80	4	100	100	25	6.25

NC: normal control; LZ + DW: negative control; LZ + CM: positive control; AE: aqueous extract of *A. ampeloprasum*; CM: clomiphene citrate + metformin; DW: distilled water; LZ: letrozole; NMR: number of mated rats; NPR: number of vaginal smear-positive rats; NGR: number of gestational rats; TNP: total number of pups; ALS: average litter size; QG: quantum gestation; GI: gestation index; and FI: fertility index.

## Data Availability

The data used to support the findings of the study are available from the corresponding author upon request.
